# Deletion of Nf2 in neural crest‐derived tongue mesenchyme alters tongue shape and size, Hippo signalling and cell proliferation in a region‐ and stage‐specific manner

**DOI:** 10.1111/cpr.13144

**Published:** 2021-10-26

**Authors:** Mohamed Ishan, Guiqian Chen, Wenxin Yu, Zhonghou Wang, Marco Giovannini, Xinwei Cao, Hong‐Xiang Liu

**Affiliations:** ^1^ Regenerative Bioscience Center University of Georgia Athens GA USA; ^2^ Department of Animal and Dairy Science College of Agricultural and Environmental Sciences University of Georgia Athens GA USA; ^3^ Department of Head and Neck Surgery David Geffen School of Medicine University of California Los Angeles Los Angeles CA USA; ^4^ Department of Developmental Neurobiology St Jude Children's Research Hospital Memphis TN USA; ^5^ Present address: College of Life Sciences Zhejiang Sci‐Tech University Hangzhou China

**Keywords:** cell proliferation, Hippo signalling, mesenchyme, neural crest, Neurofibromin 2, tongue

## Abstract

**Objectives:**

The mammalian tongue develops from the branchial arches (1–4) and comprises highly organized tissues compartmentalized by mesenchyme/connective tissue that is largely derived from neural crest (NC). This study aimed to understand the roles of tumour suppressor Neurofibromin 2 (Nf2) in NC‐derived tongue mesenchyme in regulating Hippo signalling and cell proliferation for the proper development of tongue shape and size.

**Materials and methods:**

Conditional knockout (cKO) of Nf2 in NC cell lineage was generated using *Wnt1*‐*Cre* (*Wnt1*‐*Cre*/*Nf2^cKO^*). Nf2 expression, Hippo signalling activities, cell proliferation and tongue shape and size were thoroughly analysed in different tongue regions and tissue types of *Wnt1*‐*Cre*/*Nf2^cKO^* and *Cre* *^‐^/Nf2* ^*fx/fx*^ littermates at various stages (E10.5–E18.5).

**Results:**

In contrast to many other organs in which the Nf2/Hippo pathway activity restrains growth and cell proliferation and as a result, loss of Nf2 decreases Hippo pathway activity and promotes an enlarged organ development, here we report our observations of distinct, tongue region‐ and stage‐specific alterations of Hippo signalling activity and cell proliferation in *Nf2^cKO^* in NC‐derived tongue mesenchyme. Compared to *Cre* ^−^/*Nf2^fx^* ^/^ *^fx^* littermates, *Wnt1*‐*Cre*/*Nf2^cKO^* depicted a non‐proportionally enlarged tongue (macroglossia) at E12.5–E13.5 and microglossia at later stages (E15.5–E18.5). Specifically, at E12.5 *Nf2^cKO^* mutants had a decreased level of Hippo signalling transcription factor Yes‐associated protein (Yap), Yap target genes and cell proliferation anteriorly, while having an increased Yap, Yap target genes and cell proliferation posteriorly, which lead to a tip‐pointed and posteriorly widened tongue. At E15.5, loss of Nf2 in the NC lineage resulted in distinct changes in cell proliferation in different regions, that is, high in epithelium and mesenchyme subjacent to the epithelium, and lower in deeper layers of the mesenchyme. At E18.5, cell proliferation was reduced throughout the *Nf2^cKO^* tongue.

## Graphical abstract

Schematic diagram to represent the stage‐ and region‐specific roles of Nf2‐mediated Hippo signaling in the mesenchyme during tongue organogenesis. Nf2 deletion in Neural crest‐derived tongue mesenchyme resulted in distinct alterations of Yap level and cell proliferation causing non‐proportionately larger tongue during the early stages of tongue development (i.e., E12.5‐E13.5) and microglossia during the later stages of tongue development (i.e., E15.5‐E18.5).

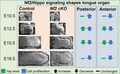

## 1 INTRODUCTION

The tongue development requires a proper regulation of its molecular mechanisms to attain its stereotypical shape, while developmental defects including microglossia, macroglossia and aglossia^1^ can hamper the normal function of the tongue, for example taste sensing, speaking and food processing. It has been shown that molecular signalling pathways and their interactions play important roles in the proper formation of the tongue.^2^, ^3^, ^4^, ^5^, ^6^, ^7^, ^8^ However, our current understanding of tongue development in relation to molecular signalling pathways is far from complete. In this study, we report the important role of Neurofibromin 2 (Nf2) in the neural crest (NC)‐derived tongue mesenchymal cells in regulating mouse tongue shape and size which is distinct from that in other organs.

In mice, the tongue forms from four branchial arches (BAs) 1–4, that is tongue primordia. Among these four BAs, BAs 1–2 give rise to the anterior two thirds of the tongue – the oral tongue, and BAs 3–4 give rise to the posterior third of the tongue – the pharyngeal tongue.^9^ The tongue emerges as three lingual swellings, two lateral and one posterior (tubercula impar) on the floor of the mandible at embryonic day (E) 11.5.^10^ These swellings fuse and grow into a spatulate tongue at E12.5. Thereafter, the tongue organ continues to grow and various types of cells are differentiated and highly organized, including formed appendages such as taste papillae and taste buds.

Nf2 is considered as a tumour suppressor gene that activates the Hippo signalling pathway through its gene product known as merlin.^11^ Under normal cellular conditions, merlin recruits mammalian sterile 20‐like protein kinase (Mst1/2), a large tumour suppressor (Lats1/2) and adaptor protein Salvador (Salv) to activate the Hippo signalling pathway by phosphorylating the transcriptional activator Yes‐associated protein (Yap).^12^, ^13^ Phosphorylation causes Yap to remain in the cytoplasm and prevents the transcription of proliferation‐specific genes.^14^ On the other hand, the absence of *Nf2* results in the lack of merlin, hence prevents Yap from phosphorylation and promotes the nuclear translocation of Yap to transcribe cell proliferation‐specific genes.^14^ Deficiencies in Nf2 function and Hippo signalling activity lead to excess cell proliferation, organ overgrowth, tumourigenesis^15^, ^16^ and metastasis,^17^ which are often promoted by the nuclear translocation of Hippo signalling repressor form, Yap protein.^18^, ^19^, ^20^

However, our results indicated paradoxically distinct roles of Nf2 in regulating Hippo signalling and cell proliferation in different regions of tongue organ at different stages. In *Wnt1*‐*Cre* driven conditional knockout of *Nf2* (*Wnt1*‐*Cre*/*Nf2^cKO^*) mice, Hippo signalling activities and cell proliferation were altered differently in anterior versus posterior tongue at early (E11.5–E13.5, macroglossia) versus late (E15.5–E18.5, microglossia) embryonic stages. In combination with previously reported data in the literature, our results indicate tissue context‐specific roles of Nf2 in regulating Hippo signalling, cell proliferation and shape and size of organs.

## 2 RESULTS

### 2.1 The immunosignals of NF2 and Yap are primarily distributed in the tongue mesenchyme

**FIGURE 1:**
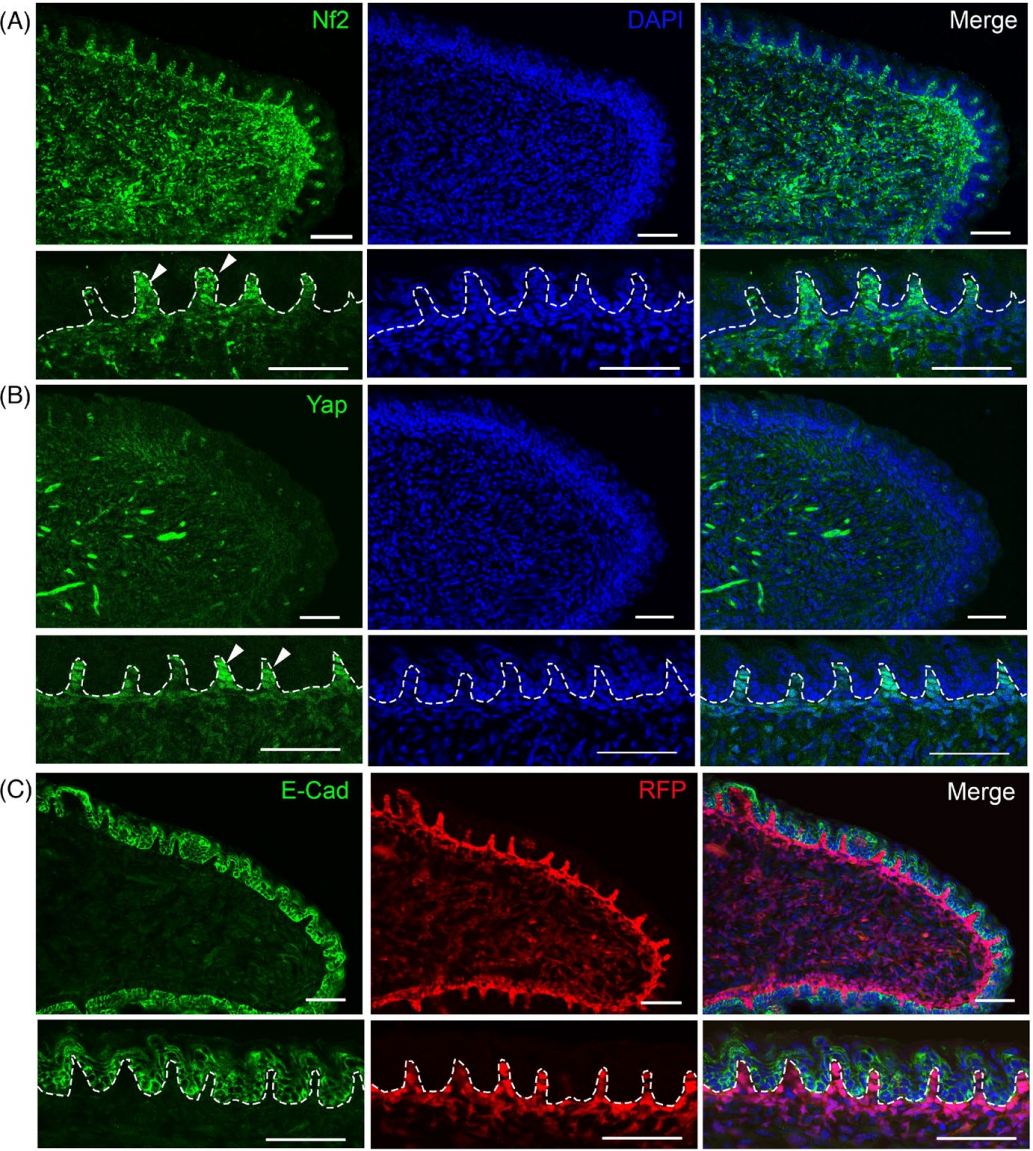
Single‐plane laser scanning confocal images of E18.5 tongue sections immunoreacted with Nf2 (A, green), Hippo signalling transcription factor Yap (B, green) or epithelial cell marker E‐cadherin (C, green). RFP^+^ cells in C were labelled by *Wnt1*‐*Cre*. Sections were counterstained with DAPI (blue). Arrowheads in A and B point to the mesenchymal layer immediately under the epithelium. Dashed lines separate the epithelium from the underlying mesenchyme. Scale bars: 50 μm

Neurofibromin 2 (Nf2) is expressed in migrating neural crest cells^21^ that give rise to a large population, if not all, of the lingual mesenchyme.^6^, ^22^ To understand where *Nf2* plays its regulatory role during tongue development, we examined the distribution of Nf2 and Yap proteins. At E18.5, Nf2 immunosignals were extensively distributed in the tongue mesenchyme (Figure 1A), but not apparent in the tongue epithelium (Figure 1A). Yap immunoproducts were broadly detected in the mesenchyme including the papilla core region subjacent to the tongue epithelium and bright signals were observed in the deeper layers of the mesenchyme of E18.5 tongue (Figure 1B). The concurrent distribution of Nf2 (bright) and Yap (faint) immunosignals was evident in the mesenchyme including the mesenchymal zone immediately under the epithelium (arrowheads in Figure 1A,B).

*Wnt1*‐*Cre* has been widely used to label NC cell lineage^4^ and labelled cells are largely distributed in the tongue mesenchyme,^6^, ^23^, ^24^ though rare labelled cells have also been found in the tongue epithelium.^6^ We have previously reported that *Wnt1*‐*Cre*‐labelled NC‐derived cells populate the mesenchyme of tongue primordium – all four branches 1–4 at E10.5.^2^To confirm *Wnt1*‐*Cre*‐driven genetic alterations in neural crest‐derived tongue mesenchyme at later stages of tongue development, E18.5 *Wnt1*‐*Cre*/*RFP* tongues were analysed and we found that RFP^+^ cells were dominantly detected in the tongue mesenchyme and no RFP^+^ cells were seen in the tongue epithelium in the examined serial sections (Figure 1C).

### 2.2 *Wnt1‐Cre* induces deletion of Nf2 in the tongue mesenchyme and reduced Nf2 transcripts in the epithelium

**FIGURE 2:**
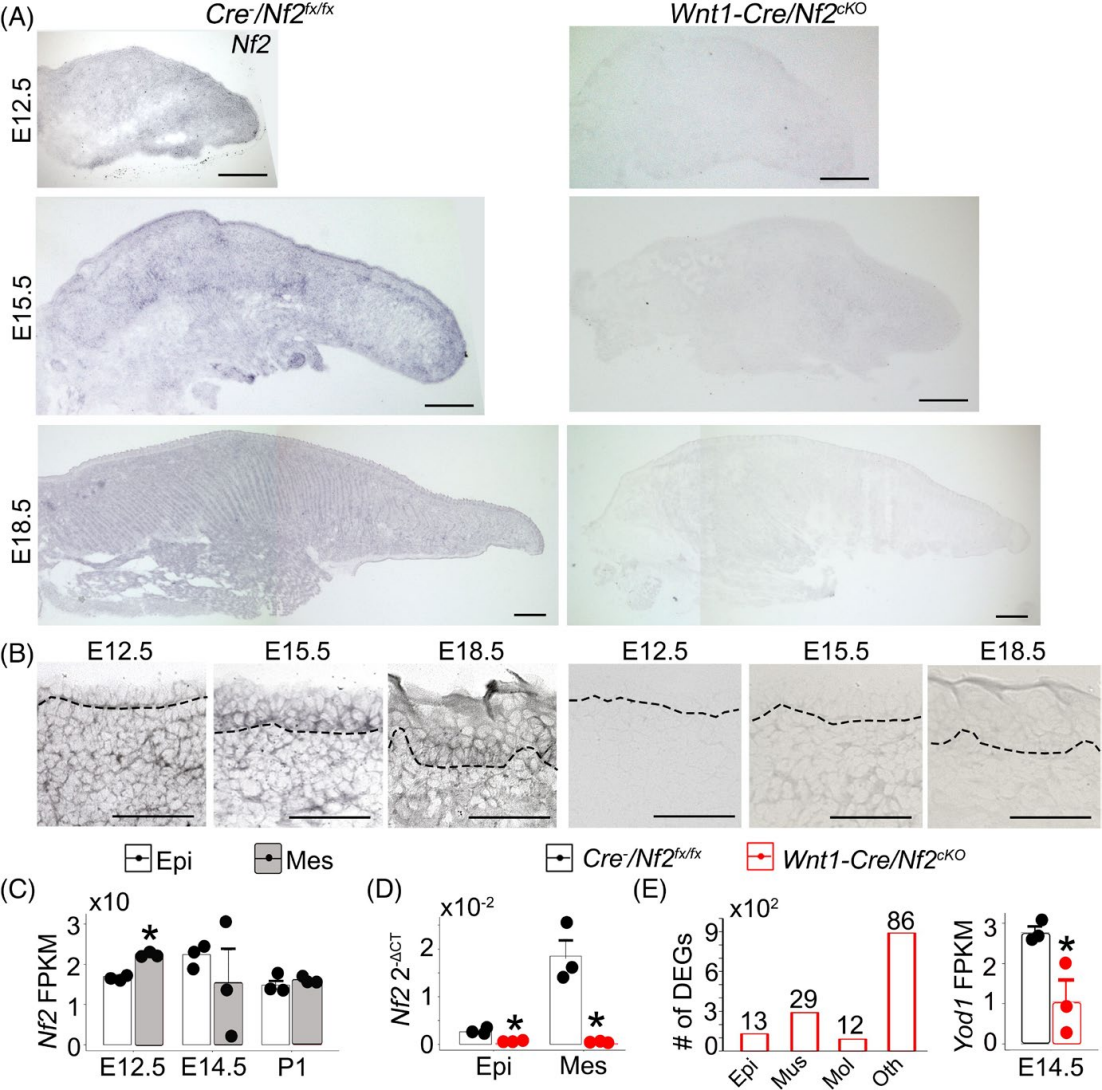
Light microscopy images of E12.5, E15.5 and E18.5 tongue sections from *Cre* ^−^/*Nf2^fx^* ^/^ *^fx^* littermate control and *Wnt1*‐*Cre*/*Nf2^cKO^* mutants at a low (A) or high (B) magnification. In situ hybridization was performed using an antisense probe for *Nf2*. Dashed lines in B separate the epithelium from the mesenchyme. Scale bars: 100 µm in A; 25 µm in B. (C‐D) Histograms (X±SD; n=3) to present the *Nf2* transcripts level in tongue epithelium (Epi) and mesenchyme (Mes), that is fragments per kilobase of transcripts per million mapped reads (FPKM) in wild‐type (C) or 2^−ΔCT^ values (D) in *Cre* ^−^/*Nf2^fx^* ^/^ *^fx^* and *Wnt1*‐*Cre*/*Nf2^cKO^* mutants. *p ≤ 0.05 compared to the corresponding tissues of *Cre* ^−^/*Nf2^fx^* ^/^ *^fx^* littermate control using two‐way ANOVA followed by Fisher's least significant difference (LSD) analyses. (E) Histograms to present the number of differentially expressed genes (DEGs) associated with Epi, muscle (Mus), molecular signalling (Mol) and other (Oth) functions (left), and FPKM (X±SD; n=3) values of Hippo signalling target gene *Yod1* (right) in E14.5 *Cre* ^−^/*Nf2^fx^* ^/^ *^fx^* and *Wnt1*‐*Cre*/*Nf2^cKO^* mutant tongues. *p ≤ 0.05 Student's *t*‐test compared to *Cre* ^−^/*Nf2^fx^* ^/^ *^fx^* littermate control group

*Nf2* in situ hybridization analyses revealed that mRNA transcripts were broadly distributed in both epithelium and mesenchyme of *Cre* ^−^/*Nf2^fx^* ^/^ *^fx^* littermate control tongues at all three stages tested (E12.5, E15.5 and E18.5) (Figure 2A, B). In addition, our RNA sequencing data also showed that *Nf2* mRNA transcripts were present in both epithelium and mesenchyme in E12.5, E14.5 and postnatal day 1 (P1) tongues with a significant difference at E12.5 when tongue mesenchyme had a higher level of *Nf2* mRNA transcripts compared to the epithelium (*p *< 0.05 in Figure 2C). In *Wnt1*‐*Cre*/*Nf2^cKO^* mutants, *Nf2* transcripts were significantly reduced in both epithelium and mesenchyme at all three stages tested (Figure 2A,B). Quantitative RT‐PCR analyses confirmed the significantly low *Nf2* expression in both epithelium and mesenchyme of E12.5 *Wnt1*‐*Cre*/*Nf2^cKO^* mutant tongues compared to that of *Cre* ^−^/*Nf2^fx^* ^/^ *^fx^* littermate controls (*p *< 0.05 in Figure 2D).

To understand the changes of genetic profile that resulted from mesenchymal *Nf2* deletion, RNA‐Seq analyses were performed for a comparison between *Wnt1*‐*Cre*/*Nf2^cKO^* mutant tongue versus *Cre* ^−^/*Nf2^fx^* ^/^ *^fx^* littermates at E14.5 (*n* = 3). A total of 140 differentially expressed genes (DEGs) were detected by Cuffdiff in the *Wnt1*‐*Cre*/*Nf2^cKO^* versus *Cre* ^−^/*Nf2^fx^* ^/^ *^fx^* littermate controls (|FC| >1, *p* < 0.05, FDR *q* < 0.05). GO terms of the DEGs showed that 13 DEGs were relevant to epithelial development and cell behaviour (Figure 2E), 29 DEGs related to muscle. In addition, 12 DEGs that were associated with multiple molecular signalling pathways, including Shh (1 DEG), Wnt (4 DEGs), FGF (2 DEGs), Rho (1 DEG) and Jak (3 DEGs) (Figure 2E). Furthermore, down‐regulation of Hippo signalling ‐regulator gene *Yod1* was detected in the *Wnt1*‐*Cre*/*Nf2^cKO^* mutant tongue compared to the *Cre* ^−^/*Nf2^fx^* ^/^ *^fx^* littermate control tongue (Figure 2E).

### 2.3 *Nf2 cKO* in neural crest‐derived tongue mesenchyme leads to macroglossia at early but microglossia at late stages of tongue development

**FIGURE 3:**
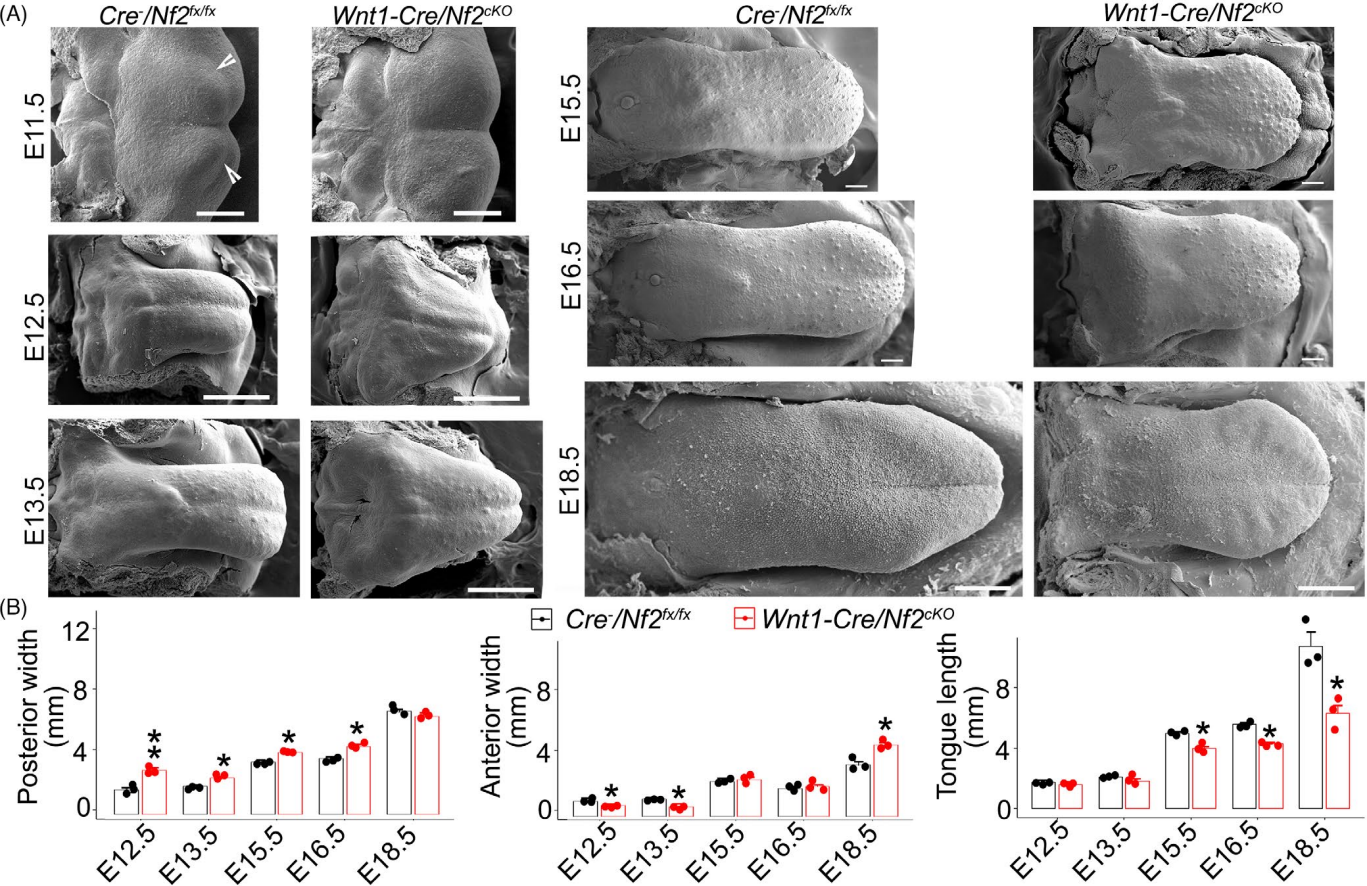
(A) Scanning electron microscopy (SEM) images of tongues on mandible in *Cre* ^−^/*Nf2^fx^* ^/^ *^fx^* littermate control and *Wnt1*‐*Cre*/*Nf2^cKO^* embryos at E11.5, E12.5, E13.5, E15.5, E16.5 and E18.5. Open arrowheads point to tongue swellings. Scale bars: 200 µm. (B) Histograms (X±SD; n=3) to present the width of the anterior and posterior oral tongues and length of the oral tongue in *Cre* ^−^/*Nf2^fx^* ^/^ *^fx^* littermate controls and *Wnt1*‐*Cre*/*Nf2^cKO^* mutants. *p ≤ 0.05, **p ≤ 0.01 Student's *t*‐test compared to *Cre* ^−^/*Nf2^fx^* ^/^ *^fx^* littermate control group

To understand the roles of Nf2 in the tongue development, phenotypic analyses were performed in *Wnt1*‐*Cre*/*Nf2^cKO^* mutant tongues and *Cre* ^−^/*Nf2^fx^* ^/^ *^fx^* littermate controls at multiple embryonic stages. At E11.5 when tongue swellings emerged from the branchial arches, tongue swellings were clearly seen in *Cre* ^−^/*Nf2^fx^* ^/^ *^fx^* littermate controls (Figure 3A). In *Wnt1*‐*Cre*/*Nf2^cKO^* mutants, tongue swellings were less profound compared to the *Cre* ^−^/*Nf2^fx^* ^/^ *^fx^* littermates (Figure 3A).

At E12.5 and E13.5 (Figure 3A), tongue shape and size in *Wnt1*‐*Cre*/*Nf2^cKO^* mutants were altered significantly compared to *Cre* ^−^/*Nf2^fx^* ^/^ *^fx^* littermate controls (Figure 3A). The *Wnt1*‐*Cre*/*Nf2^cKO^* mutant tongues were significantly narrower in the anterior oral tongue region and significantly wider towards the posterior oral tongue region (Figure 3A–B; *p *< 0.05) compared to that of *Cre* ^−^/*Nf2^fx^* ^/^ *^fx^* littermates (Figure 3). Even though the length of the oral tongue was not significantly altered in the *Wnt1*‐*Cre*/*Nf2^cKO^* mutants (Figure 3A–B; *p *> 0.05), increase in the tongue width resulted in an overall enlarged tongue (i.e. macroglossia) in *Wnt1*‐*Cre*/*Nf2^cKO^* mutants compared to *Cre* ^−^/*Nf2^fx^* ^/^ *^fx^* littermate control (Figure 3).

At E15.5‐E16.5, *Wnt1*‐*Cre*/*Nf2^cKO^* mutants continued to develop a significantly wider posterior oral tongue (Figure 3A‐B; *p *< 0.05) compared to that of *Cre* ^−^/*Nf2^fx^* ^/^ *^fx^* littermates (Figure 3). In contrast to the early stages (i.e. E12.5 and E13.5), the E15.5–E16.5 oral tongues of *Wnt1*‐*Cre*/*Nf2^cKO^* mutants were significantly shorter (Figure 3A‐B; *p *< 0.05) than those of *Cre* ^−^/*Nf2^fx^* ^/^ *^fx^* littermates (Figure 3). No significant differences in anterior oral tongue width were noticed between *Wnt1*‐*Cre*/*Nf2^cKO^* mutants (Figure 3A, B; *p *> 0.05) and *Cre* ^−^/*Nf2^fx^* ^/^ *^fx^* littermates (Figure 3A). As a result of the collective changes in the tongue length and width, E15.5–E16.5 *Wnt1*‐*Cre*/*Nf2^cKO^* mutants had a relatively smaller tongue (i.e. microglossia, Figure 3A) compared to the *Cre* ^−^/*Nf2^fx^* ^/^ *^fx^* littermates (Figure 3A).

At E18.5, microglossia phenotype in the *Wnt1*‐*Cre*/*Nf2^cKO^* mutants was evident compared to *Cre* ^−^/*Nf2^fx^* ^/^ *^fx^* littermates (Figure 3A). *Wnt1*‐*Cre*/*Nf2^cKO^* mutant mice had a significantly shorter oral tongue compared to those of *Cre* ^−^/*Nf2^fx^* ^/^ *^fx^* littermate controls (Figure 3A,B; *p *< 0.05). Unlike the earlier stages (E12.5–E16.5), no significant changes in the width of posterior oral tongue were observed in the *Wnt1*‐*Cre*/*Nf2^cKO^* mutants compared to *Cre* ^−^/*Nf2^fx^* ^/^ *^fx^* littermates (Figure 3A,B; *p *> 0.05).

### 2.4 Region‐ and stage‐specific alterations of Hippo signalling in *Wnt1‐Cre/Nf2 cKO* mouse tongues

Nf2 is a known cell proliferation suppressor and often acts via Hippo‐YAP signalling to regulate organ size.^13^, ^25^ To understand the potential cause of the alteration of tongue shape and size, we examined the Hippo signalling activity in different tongue regions at different stages of *Wnt1*‐*Cre*/*Nf2^cKO^* mutants and *Cre* ^−^/*Nf2^fx^* ^/^ *^fx^* littermate controls.

**FIGURE 4:**
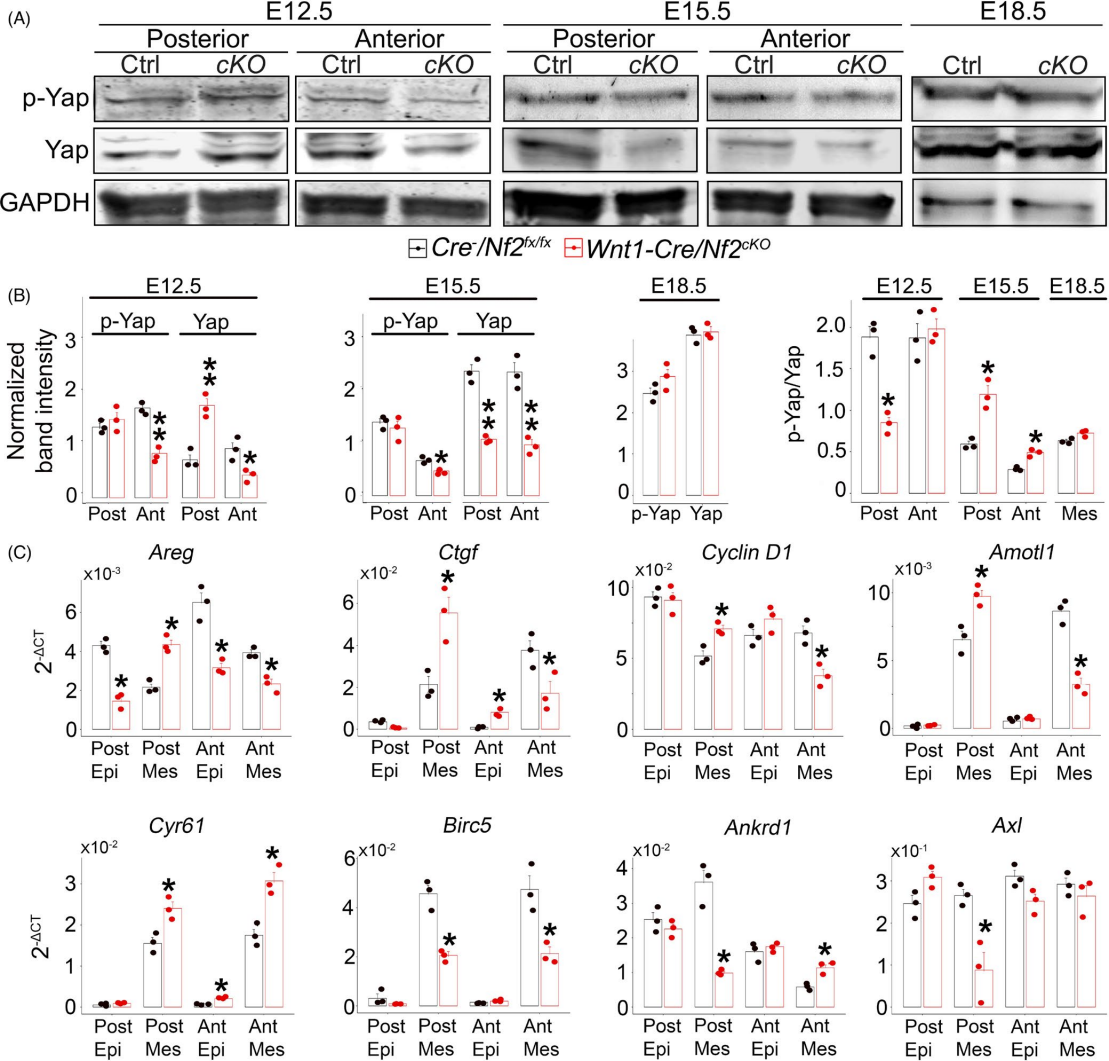
(A) Western blot bands of p‐Yap, Yap and GAPDH in the E12.5, E15.5 and E18.5 tongue mesenchyme from the anterior (Ant) and posterior (Post) oral tongues (E12.5 and E15.5) or the entire tongue (E18.5) of *Cre* ^−^/*Nf2^fx^* ^/^ *^fx^* (Ctrl) and *Wnt1*‐*Cre*/*Nf2^cKO^* (*cKO*) embryos. (B) Histograms (X ± SD; *n* = 3) to present the normalized band intensities of p‐Yap and Yap relative to the GAPDH; and p‐Yap/Yap ratios in the E12.5, E15.5 and E18.5 tongue mesenchyme. *p ≤ 0.05, **p ≤ 0.01 Student's *t*‐test compared to *Cre* ^−^/*Nf2^fx^* ^/^ *^fx^* littermate control group. (C) Histograms (X±SD; n=3) to present the 2^−ΔCT^ values of Yap target genes in posterior and anterior tongue epithelium (Epi) and mesenchyme (Mes) of E12.5 *Cre* ^−^/*Nf2^fx^* ^/^ *^fx^* and *Wnt1*‐*Cre*/*Nf2^cKO^* mutants. *p ≤ 0.05, **p ≤ 0.01 compared to *Cre* ^−^/*Nf2^fx^* ^/^ *^fx^* littermate control using two‐way ANOVA followed by Fisher's least significant difference (LSD) analyses

Western blot analyses on downstream signalling components of Hippo pathway revealed that at E12.5 the deactivated form of transcriptional regulator p‐Yap was at a significantly lower level in the anterior (Figure 4A,B; *p *< 0.05), but not posterior (Figure 4A,B; *p *> 0.05), oral tongue mesenchyme of *Wnt1*‐*Cre*/*Nf2^cKO^* mutants compared to the corresponding regions of the *Cre* ^−^/*Nf2^fx^* ^/^ *^fx^* littermate control. In contrast to p‐Yap, the transcriptional activator of Hippo signalling pathway Yap that promotes cell proliferation was at a significantly higher level in the posterior, however lower in the anterior, oral tongue mesenchyme in *Wnt1*‐*Cre*/*Nf2^cKO^* mutant compared to the corresponding tongue regions of the *Cre* ^−^/*Nf2^fx^* ^/^ *^fx^* littermates (Figure 4A,B; *p *< 0.05).

At E15.5, the anterior oral tongue mesenchyme of *Wnt1*‐*Cre*/*Nf2^cKO^* mutants had a significantly lower level of p‐Yap than the corresponding region of the *Cre* ^−^/*Nf2^fx^* ^/^ *^fx^* littermates (Figure 4A,B; *p *< 0.05). However, no significant changes of p‐Yap level were detected in the posterior oral tongue mesenchyme of E15.5 *Wnt1*‐*Cre*/*Nf2^cKO^* mutants compared to *Cre* ^−^/*Nf2^fx^* ^/^ *^fx^* littermates (Figure 4A,B; *p *> 0.05). In contrast to p‐Yap, Yap was detected at a significantly lower level in both posterior and anterior oral tongue mesenchyme of E15.5 *Wnt1*‐*Cre*/*Nf2^cKO^* mutants compared to the respective regions of *Cre* ^−^/*Nf2^fx^* ^/^ *^fx^* littermates (Figure 4A,B; *p *< 0.01). At E18.5, no significant changes of p‐Yap and Yap level were detected in the tongue mesenchyme of *Wnt1*‐*Cre*/*Nf2^cKO^* mutants compared to *Cre* ^−^/*Nf2^fx^* ^/^ *^fx^* littermates (Figure 4A,B; *p *> 0.05).

The ratio of p‐Yap/Yap was calculated to represent the Hippo pathway activity. The p‐Yap/Yap ratio was altered distinctly in different tongue regions at different stages in *Wnt1*‐*Cre*/*Nf2^cKO^* mutants compared to the *Cre* ^−^/*Nf2^fx^* ^/^ *^fx^* littermates. A significantly lower ratio of p‐Yap/Yap was detected in the posterior, but not anterior, E12.5 tongue mesenchyme (Figure 4B). At E15.5, a significantly higher p‐Yap/Yap ratio was detected in both posterior and anterior tongue mesenchyme of the *Wnt1*‐*Cre*/*Nf2^cKO^* mutants (Figure 4B). No significant changes in p‐Yap/Yap ratio were found in E18.5 *Wnt1*‐*Cre*/*Nf2^cKO^* mutant tongue mesenchyme (Figure 4B).

Quantitative RT‐PCR analyses on Yap target genes that are known regulators of cell proliferation in E12.5 *Wnt1*‐*Cre*/*Nf2^cKO^* and *Cre* ^−^/*Nf2^fx^* ^/^ *^fx^* tongues revealed region‐ and tissue‐specific changes in gene expression levels. Among the genes tested, distinct expression patterns with respect to tongue regions (anterior vs. posterior) and tissue types (epithelium vs. mesenchyme) were found (Figure 4C).

In the mesenchyme of E12.5 tongues of *Wnt1*‐*Cre*/*Nf2^cKO^* mutants compared to the *Cre* ^−^/*Nf2^fx^* ^/^ *^fx^* littermate controls: (1) *Areg*, *Ctgf*, *Cyclin D1* and *Amotl1* expression levels were significantly up‐regulated in the posterior, and on the contrary, down‐regulated in the anterior, oral tongue mesenchyme (*p *< 0.05); (2) *Ankrd1* expression was significantly down‐regulated in the posterior, but up‐regulated in the anterior oral tongue mesenchyme (*p* < 0.05); (3) *Cyr61* expression was significantly up‐regulated, while *Birc5* down‐regulated in both posterior and anterior oral tongue mesenchyme (*p *< 0.05); (4) *Axl* gene expression was significantly down‐regulated in the posterior (*p * < 0.05), but not altered in the anterior (*p* > 0.05) tongue mesenchyme.

In the epithelium of E12.5 tongues of *Wnt1*‐*Cre*/*Nf2^cKO^* mutants compared to the *Cre* ^−^/*Nf2^fx^* ^/^ *^fx^* littermate controls, of all eight genes tested, *Areg*, *Ctgf and Cyr61* had significant alterations in gene expression levels (Figure 4C), that is *Areg* expression levels were significantly lower in both posterior and anterior tongue epithelium, while *Ctgf* and *Cyr61* expression were significantly up‐regulated in the anterior oral tongue epithelium (*p* <0.05).

### 2.5 Region‐ and stage‐specific alterations of cell proliferation in *Wnt1‐Cre/Nf2 cKO* mouse tongues

To understand the effects of altered Hippo signalling activity in the *Wnt1*‐*Cre*/*Nf2^cKO^* mutant mesenchyme and the cause of tongue shape/size alterations, cell proliferation was analysed in *Wnt1*‐*Cre*/*Nf2^cKO^* mutants at multiple stages. We found distinct alterations in different tongue regions at different stages.

**FIGURE 5:**
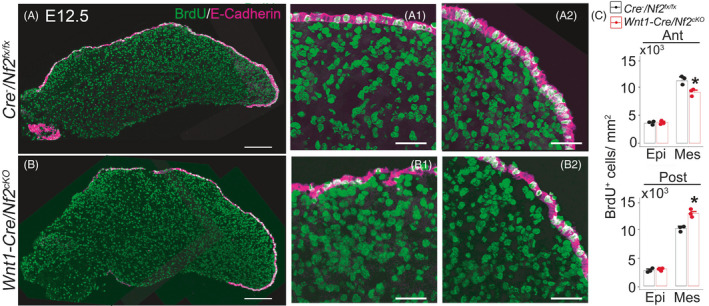
Single‐plane laser scanning confocal images of tongue sections in E12.5 *Cre* ^−^/*Nf2^fx^* ^/^ *^fx^* littermate control (A) and *Wnt1*‐*Cre*/*Nf2^cKO^* (B) mutants. Sections were immunoreacted with S‐phase cell proliferation marker BrdU (green) and epithelial cell marker E‐cadherin (magenta). A_1–2_ and B_1–2_ are high magnification images of the posterior (A_1_, B_1_) and anterior (A_2_, B_2_) oral tongue regions. Scale bars: 200 μm in A, B; 50 μm in A_1–2_, B_1–2_. (C) Histograms (X±SD; n=3) to present the number of BrdU^+^ cells per mm^2^ in the anterior (Ant) and posterior (Post) oral tongues of *Cre* ^−^/*Nf2^fx^* ^/^ *^fx^* littermate control and *Wnt1*‐*Cre*/*Nf2^cKO^* mutants. *p ≤ 0.05 compared to the corresponding regions of *Cre* ^−^/*Nf2^fx^* ^/^ *^fx^* littermate control using two‐way ANOVA followed by Fisher's LSD analyses

At E12.5 (Figure 5), more BrdU^+^ cells were observed in the posterior (*p *< 0.05), however fewer in the anterior (*p *< 0.05), oral tongue mesenchyme of *Wnt1*‐*Cre*/*Nf2^cKO^* mutants (Figure 5B) compared to the *Cre* ^−^/*Nf2^fx^* ^/^ *^fx^* littermate control (Figure 5A). No obvious changes (*p *> 0.05) in the number of BrdU^+^ cells were seen in the tongue epithelium of E12.5 *Wnt1*‐*Cre*/*Nf2^cKO^* mutants (Figure 5B) compared to *Cre* ^−^/*Nf2^fx^* ^/^ *^fx^* littermates (Figure 5A).

**FIGURE 6:**
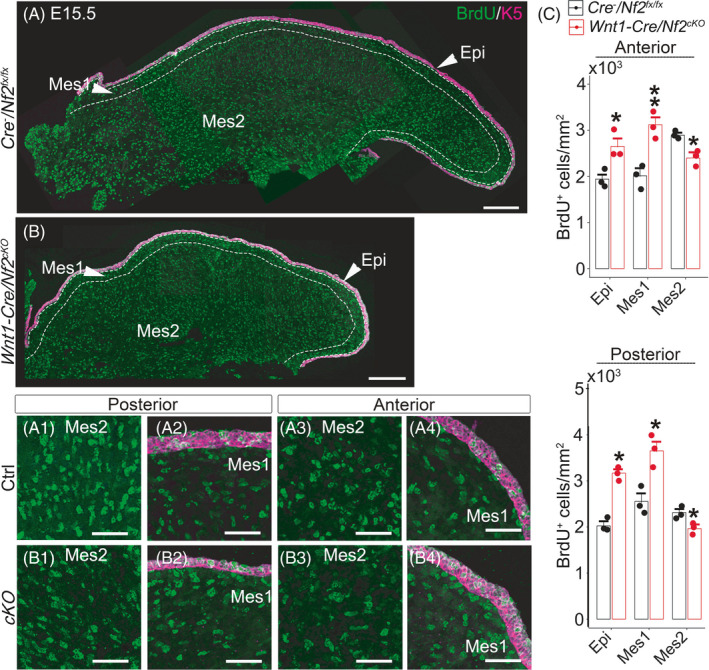
Single‐plane laser scanning confocal images of tongue sections in E15.5 *Cre* ^−^/*Nf2^fx^* ^/^ *^fx^* littermate control (Ctrl, A) and *Wnt1*‐*Cre*/*Nf2^cKO^* (*cKO*, B) mutants. Sections were immunoreacted with S‐phase cell proliferation marker BrdU (green) and epithelial cell marker Krt5 (magenta). A_1–4_ and B_1–4_ are high magnification images of the posterior (A_1–2_, B_1–2_) and anterior (A_3–4_, B_3–4_) oral tongue regions. Epi: epithelium (A_2_, B_2_ and A_4_, B_4_); Mes1: mesenchyme layer just beneath the epithelium (A_2_, B_2_ and A_4_, B_4_); Mes2: deeper mesenchymal layers (A_1_, B_1_ and A_3_, B_3_) of the posterior (A_1_, B_1_) and anterior (A_3_, B_3_) oral tongues. Dashed lines in A and B demarcate the borders between Epi, Mes1 and Mes2. Scale bars: 200 μm in A, B; 50 μm in A_1–4_, B_1–4_. (C) Histograms (X±SD; n=3) to present the number of BrdU^+^ cells per mm^2^ in the anterior and posterior oral tongue epithelium and mesenchyme of *Cre* ^−^/*Nf2^fx^* ^/^ *^fx^* littermate control and *Wnt1*‐*Cre*/*Nf2^cKO^* mutants. *p ≤ 0.05, **p ≤ 0.01 compared to *Cre* ^−^/*Nf2^fx^* ^/^ *^fx^* littermate control using two‐way ANOVA followed by Fisher's LSD analyses

At E15.5 (Figure 6), in *Wnt1*‐*Cre*/*Nf2^cKO^* mutants as compared to the corresponding regions of the *Cre* ^−^/*Nf2^fx^* ^/^ *^fx^* littermates, a significantly higher amount of BrdU^+^ cells were detected in the epithelium (*p *< 0.05) and mesenchyme just beneath the epithelium (*p* < 0.05) of both posterior (Figure 6B2) and anterior (Figure 6B4) oral tongue. In contrast, a significantly lower amount of BrdU^+^ cells were detected in the deeper mesenchyme layers of both posterior (Figure 6B1; *p* < 0.05) and anterior (Figure 6B3; *p *<  0.05) oral tongue (Mes2 in Figure 6B).

**FIGURE 7:**
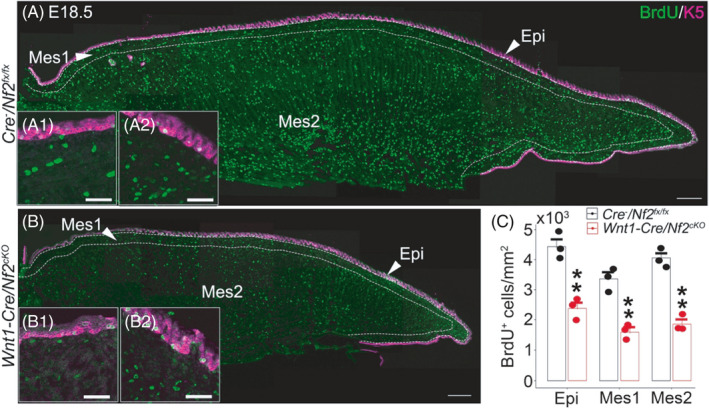
Single‐plane laser scanning confocal images of tongue sections in E18.5 *Cre* ^−^/*Nf2^fx^* ^/^ *^fx^* littermate control (A) and *Wnt1*‐*Cre*/*Nf2^cKO^* mutants (B). Sections were immunoreacted with S‐phase cell proliferation marker BrdU (green) and epithelial cell marker Krt5 (magenta). Insets A_1–2_ and B_1–2_ are high magnification images of the posterior (A_1_, B_1_) and anterior (A_2_, B_2_) oral tongue regions. Epi: epithelium; Mes1: mesenchyme layer just beneath the epithelium; Mes2: deeper mesenchymal layers. Dashed lines in A and B demarcate the borders between Epi, Mes1 and Mes2. Scale bars: 200 μm in A, B; 50 μm in A_1–4_, B_1–4_. (C) Histogram (X±SD; n=3) to present the number of BrdU^+^ cells per mm^2^ in the tongue epithelium and mesenchyme of *Cre* ^−^/*Nf2^fx^* ^/^ *^fx^* littermate control and *Wnt1*‐*Cre*/*Nf2^cKO^* mutants. **p ≤ 0.01 compared to the corresponding regions of *Cre* ^−^/*Nf2^fx^* ^/^ *^fx^* littermate control using two‐way ANOVA followed by Fisher's LSD analyses

At E18.5, in *Wnt1*‐*Cre*/*Nf2^cKO^* mutants (Figure 7B) compared to the *Cre* ^−^/*Nf2^fx^* ^/^ *^fx^* littermate controls (Figure 7A, A1, and A2), a significantly lower number of BrdU^+^ cells (*p* < 0.01) was detected in all tongue regions and tissue types, including epithelium and mesenchyme in both posterior (Figure 7B1) and anterior (Figure 7B2) oral tongue regions.

### 2.6 Distinct alterations of cell proliferation in different regions of tongue primordium (branchial arches) in mesenchymal *Nf2 cKO* mice

To understand the potential cause of the unproportioned alterations in tongue shape and size at early stages, we examined the Nf2 expression, Hippo signalling activity and cell proliferation in E10.5 branchial arches (BAs) 1–4 of *Wnt1*‐*Cre*/*Nf2^cKO^* mutants and *Cre* ^−^/*Nf2^fx^* ^/^ *^fx^* littermate controls.

**FIGURE 8:**
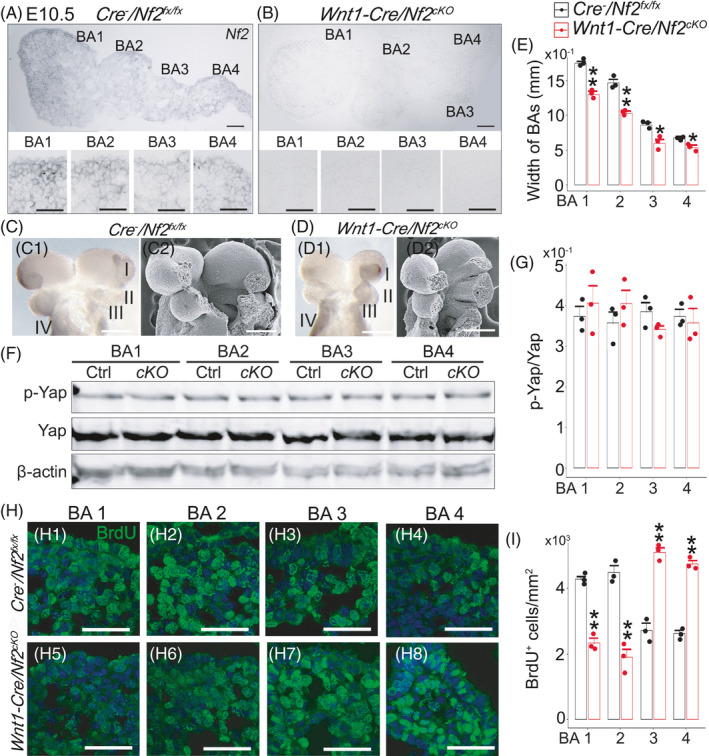
Light microscopy images of sections of E10.5 branchial arches (BAs) from a *Cre* ^−^/*Nf2^fx^* ^/^ *^fx^* littermate control (Ctrl, A) and *Wnt1*‐*Cre*/*Nf2^cKO^* mutant (*cKO*, B). In situ hybridization was performed using an antisense probe for *Nf2*. Bottom panel in A and B is high magnification images of the individual BAs. Scale bars: 25 µm. Whole‐mount images (bright field in C_1_, D_1_, and SEM in C_2_, D_2_) of the *Cre* ^−^/*Nf2^fx^* ^/^ *^fx^* littermate control (C) and *Wnt1*‐*Cre*/*Nf2^cKO^* (D) BAs. Roman numerals I–IV in C_1_ and D_1_ represent BAs 1–4. Scale bars: 200 μm. (E) Histogram (X±SD; n=3) to present the width of each BA (between the lateral edges) in *Cre* ^−^/*Nf2^fx^* ^/^ *^fx^* littermate controls and *Wnt1*‐*Cre*/*Nf2^cKO^* mutants. *p ≤ 0.05, **p ≤ 0.01 compared to the corresponding BA of *Cre* ^−^/*Nf2^fx^* ^/^ *^fx^* littermate control using two‐way ANOVA followed by Fisher's LSD analyses. (F) Western blot bands of p‐Yap, Yap and β‐actin in the mesenchyme of individual BAs from *Cre* ^−^/*Nf2^fx^* ^/^ *^fx^* littermate controls and *Wnt1*‐*Cre*/*Nf2^cKO^* mutants. (G) Histograms (X±SD; n=3) to present p‐Yap/Yap ratio in the mesenchyme of individual BAs from *Cre* ^−^/*Nf2^fx^* ^/^ *^fx^* littermate controls and *Wnt1*‐*Cre*/*Nf2^cKO^* mutants. No statistical significance was detected in *Wnt1*‐*Cre*/*Nf2^cKO^* mutants compared to *Cre* ^−^/*Nf2^fx^* ^/^ *^fx^* littermate control group. (H) Single‐plane laser scanning confocal images of sagittal BA sections immunostained with S‐phase cell proliferation marker BrdU (green) in *Cre* ^−^/*Nf2^fx^* ^/^ *^fx^* littermate control (H_1‐4_) and *Wnt1*‐*Cre*/*Nf2^cKO^* mice (H_5–8_). Scale bars: 50 μm. (I) Histograms (X±SD; n=3) to present the number of BrdU^+^ cells per mm^2^ in individual BAs of *Cre* ^−^/*Nf2^fx^* ^/^ *^fx^* littermate controls and *Wnt1*‐*Cre*/*Nf2^cKO^* mutants. **p ≤ 0.01 compared to the corresponding BA of *Cre* ^−^/*Nf2^fx^* ^/^ *^fx^* littermate control using two‐way ANOVA followed by Fisher's LSD analyses

*Nf2* in situ hybridization revealed that in *Cre* ^−^/*Nf2^fx^* ^/^ *^fx^* littermate controls *Nf2* mRNA transcripts were abundantly distributed in all four BAs that will give rise to the developing tongue (Figure 8A). In *Wnt1*‐*Cre*/*Nf2^cKO^* mutants, *Nf2* transcripts were absent in the corresponding regions of all four BAs (Figure 8B). Similar to *Cre* ^−^/*Nf2^fx^* ^/^ *^fx^* littermate controls (Figure 8C), all four BAs (n = 3) were developed in the *Wnt1*‐*Cre*/*Nf2^cKO^* mutants (Figure 8D). However, the distance between the lateral edges of each BA was significantly smaller in *Wnt1*‐*Cre*/*Nf2^cKO^* mutants (Figure 8D,E, *p* < 0.05) compared to those of *Cre* ^−^/*Nf2^fx^* ^/^ *^fx^* littermate controls (Figure 8C).

The downstream transcriptional regulators of Hippo signalling, that is Yap and p‐Yap, were both detected in the *Wnt1*‐*Cre*/*Nf2^cKO^* and *Cre* ^−^/*Nf2^fx^* ^/^ *^fx^* BAs (Figure 8F). No significant differences in Western blot band intensities of p‐Yap, Yap and p‐Yap/Yap ratios were detected between the BAs of *Cre* ^−^/*Nf2^fx^* ^/^ *^fx^* littermate control and those of *Wnt1*‐*Cre*/*Nf2^cKO^* mutants (Figure 8F,G; *p *> 0.05).

Cell proliferation was altered distinctly in different BAs. BAs 1–2 of *Wnt1*‐*Cre*/*Nf2^cKO^* mutants had a significantly lower, while BAs 3–4 had a significantly higher number of BrdU^+^ cells in *Wnt1*‐*Cre*/*Nf2^cKO^* mutants compared to the *Cre* ^−^/*Nf2^fx^* ^/^ *^fx^* littermate controls (Figure 8H,I; *p *< 0.05).

## 3 DISCUSSION

Our study demonstrated that the absence of Neurofibromin 2 (Nf2)/Merlin in the neural crest (NC) derived‐mesenchyme leads to macroglossia at early (E12.5–E13.5) and microglossia at later (E15.5–E18.5) stages of embryonic tongue development. The tongue deformity (i.e. pointed anterior oral tongue and wider posterior oral tongue) along the anteroposterior axis and distinct changes in the Hippo signalling activity accompanied by changes in cell proliferation in different regions of the *Wnt1*‐*Cre*/*Nf2^cKO^* mutant tongue indicate that NC‐derived mesenchymal cells in developing tongue respond to the absence of Nf2 in a region‐ and stage‐specific manner. Overall, our results indicated that Nf2 in NC‐derived mesenchyme plays an essential role in regulating Hippo signalling activity and cell proliferation for the proper development of tongue shape and size.

### 3.1 Nf2/Hippo signalling activity in the NC‐derived mesenchyme regulates tongue organogenesis in a stage‐specific manner

NC cells migrate into the tongue primordium (i.e. branchial arches) at early embryonic stages^2^, ^26^, ^27^ and fully occupy the mesenchyme of the tongue bud.^28^ Structural and molecular integrity of the NC and NC‐derived cells in the tongue primordium is critical for proper tongue organ development. As an example, the absence of primary cilia in NC cells causes aglossia.^8^ Deficient molecular signalling, such as BMP,^2^ TGF‐β^3^, ^29^, ^30^ and Wnt,^4^, ^5^, ^31^, ^32^ results in microglossia due to defects in structural organization and cell migration in the developing tongue. In this study, we report the importance of continuous regulation of Nf2/Hippo signalling in NC and NC‐derived tongue mesenchymal cells for proper tongue organogenesis.

Nf2, the product of the gene responsible for the disease neurofibromatosis type 2,^33^ is pivotal for embryo development, including the development of NC^34^, ^35^ and oral structures.^36^ Nf2 is well‐known as a suppressor of cell proliferation and tumour.^37^, ^38^, ^39^ In many cases, Nf2 activates Hippo signalling, an evolutionarily conserved potent regulator of cell proliferation and organ size.^13^, ^25^ The Hippo pathway is driven by a core kinase cascade that includes Mst1/2 and Lats1/2, which in turn phosphorylate and inactivate Yes‐associated protein 1 (YAP) and TAZ transcriptional co‐activators.^13^

In most organs, the absence of Nf2/Hippo signalling causes an enhanced organ growth,^40^, ^41^ that is liver‐specific genetic knockout of *Mst1*/*2*, *Sav1*, *Nf2* or over‐expression of *Yap* resulted in an enlarged liver,^18^, ^42^, ^43^, ^44^ deletion of *Sav1*, *Mst1*/*2* or *Lats1*/*2* caused a hyperproliferation and enlarged heart.^45^, ^46^, ^47^, ^48^, ^49^, ^50^ However, our data indicate that Nf2/Hippo signalling regulates tongue organogenesis in a region‐ and stage‐specific manner. The primordia (branchial arches/BAs) of the tongue organ were narrower at E10.5 and lingual swellings were less profound in *Wnt1*‐*Cre*/*Nf2^cKO^* mutants. At E12.5–E13.5, deletions of *Nf2* in NC lineage driven by *Wnt1*‐*Cre* lead to a non‐proportionally enlarged tongue, that is, macroglossia, with a widened posterior region and pointed tip. At E15.5‐E18.5, in contrast to many other organs, we observed a smaller tongue (microglossia) suggesting that the effects of altered Nf2/Hippo signalling activity are distinct in different organs.

### 3.2 Stage‐ and tongue region‐specific regulation of Hippo signalling and cell proliferation in response to mesenchymal *Nf2* deletion

As aforementioned, Nf2/Hippo signalling serves as a cell proliferation suppressor.^51^ To understand the stage‐specific alterations of tongue shape and size in *Wnt1*‐*Cre*/*Nf2^cKO^* mice, we analysed the levels of Yap and p‐Yap and cell proliferation in different tongue regions and tissue types at various stages. We detected regional and dynamic changes of Yap and p‐Yap levels over the developmental course and found that cell proliferation was altered in general corresponding to the Yap level as the Yap typically promotes cell proliferation.^18^, ^19^, ^20^

At E10.5 BAs of *Wnt1*‐*Cre*/*Nf2^cKO^* mutant and *Cre* ^−^/*Nf2^fx^* ^/^ *^fx^* littermate control mice, even though individual BAs of *Wnt1*‐*Cre*/*Nf2^cKO^* mutant mice had no significant changes of levels of Hippo signalling downstream transcriptional regulator Yap and deactivated form p‐Yap, significantly different amounts of BrdU^+^ cells suggest that cell proliferation is very sensitive and responded to subtle yet undetectable changes of Yap and p‐Yap levels in individual BAs of *Wnt1*‐*Cre*/*Nf2^cKO^* mutant. Consequently, the initial formation of lingual swellings at E11.5 was deficient on the *Wnt1*‐*Cre*/*Nf2^cKO^* mutant as a result of low amount proliferative cells in BAs1–2. It is reasonable to speculate that a low number of proliferative cells in BAs 1–2 and more proliferative cells in BAs 3–4 might be one of the reasons to have a pointed anterior tongue and widened posterior tongue since BAs 1–2 and BAs 3–4 give rise to the anterior and posterior tongue regions^9^ respectively.

Our thorough phenotypic analyses along with Hippo signalling activities in *Wnt1*‐*Cre*/*Nf2^cKO^* mutants demonstrated that continuous expression of *Nf2* in NC‐derived mesenchymal cells is required for the proper tongue formation in shape and size. At E12.5, a high Yap level and Yap target genes (*Areg*, *Ctgf*, *cyclin D1* and *Amotl1*) that are known regulators of cell proliferation were concurrent with more proliferative cells in the posterior oral tongue where the *Nf2 cKO* tongue is wider, and low Yap level with fewer proliferative cells in the anterior oral tongue where the tongue is smaller and more pointed. It is important to note that some Yap target genes (*Cyr61*, *Birc5*, *Ankrd1* and *Axl*) had different expression levels in contrast to the Yap levels detected in the E12.5 tongue suggesting that Yap might regulate the expression of different target genes distinctly.

Notably, at later stages of tongue development (E15.5–E18.5), the loss of *Nf2* in the NC lineage paradoxically resulted in decreased cell proliferation (reduced Yap level), thus resulting in a smaller tongue (i.e. microglossia) in *Wnt1*‐*Cre*/*Nf2^cKO^* mutants compared to the *Cre* ^−^/*Nf2^fx^* ^/^ *^fx^* littermates. Even though cell proliferation was reduced as a whole in the E15.5 *Wnt1*‐*Cre*/*Nf2^cKO^* mutants, different tissue compartments had significantly different numbers of BrdU^+^ cells in the *Wnt1*‐*Cre*/*Nf2^cKO^* mutants, for example the increased versus decreased cell proliferation in the shallow and deep layer of mesenchyme respectively. The increased cell proliferation in the mesenchyme layer subjacent to the epithelium represents a direct effect of Nf2 deletion on the NC‐derived mesenchymal cells as the cells populated in this region are mostly, if not all, from NC, and as a result, *Nf2 cKO* in these cells leads to an inactivation of Hippo signalling, hence promotes the cell proliferation.

With regard to the alterations of Hippo signalling (Yap level) and cell proliferation in tongue regions at late embryonic stages (E15.5–E18.5), which are not consistent with conventional Nf2/Hippo/Yap‐cell proliferation pathway, it is possible that *Nf2* deletion causes changes in other signalling pathways that overwrite the effects of diminished Hippo signalling in the tongue, for example Wnt/β‐catenin, TGF‐β, Hedgehog.^52^ Moreover, Wnt5a/non‐canonical Wnt signalling has been reported to be important for tongue outgrowth^4^ and is likely to be involved in the regulatory role of Nf2/Hippo signalling in tongue mesenchyme in promoting tongue formation. Moreover, tongue mesenchyme acts as a scaffold for the organization of migrating myoblasts into the myogenic cord and operates as a niche that releases molecular instructions (e.g. TGF‐β2) to direct survival, proliferation and differentiation of myogenic progenitors, as well as patterning of muscle fibres.^53^, ^54^ Thus, disrupted myogenesis might be another cause of microglossia observed in *Wnt1*‐*Cre*/*Nf2^cKO^* mutants at E15.5–E18.5. Indeed, our RNA‐sequencing data reveal an altered gene expression profile in muscles and multiple molecular signalling that interact with Hippo signalling.^55^, ^56^, ^57^

### 3.3 Mesenchymal deletion of *Nf2* alters the development of overlying tongue epithelium

It is intriguing that although the genetic deletion of *Nf2* driven by *Wnt1*‐*Cre* is largely tongue mesenchyme‐specific,^6^, ^24^ a lack of *Nf2* expression in the tongue epithelium was noticed in *Wnt1*‐*Cre*/*Nf2^cKO^* mice. In addition, our RNA‐seq data demonstrate that multiple DEGs related to epithelial cell organization were affected in the *Wnt1*‐*Cre*/*Nf2^cKO^* mice. Our data suggest that Nf2 regulates mesenchymal–epithelial interactions. Given that Nf2 immunoproducts are absent in the tongue epithelium in wild type mice, it is reasonable to speculate that alterations of epithelial cells (e.g. proliferation and organization) are caused by the loss of mesenchymal *Nf2*. Moreover, presence of many DEGs related to multiple molecular signalling pathways including sonic hedgehog and Wnt further support the idea that Nf2/Hippo signalling in the mesenchyme plays an important role in the mesenchymal–epithelial interactions. Future studies are important to address whether and how the development of tongue epithelial appendages including taste papillae and taste buds is affected in the mesenchymal *Nf2 cKO*.

It has been reported that *Nf2* gene mutation can cause tongue Schwannoma, a tumour originating from Schwann cells.^58^, ^59^, ^60^ These Schwannomas occur in the tongue more frequently than other regions in the oral cavity,^59^, ^61^ suggesting a region‐specific role of Nf2 in oral tumourigenesis. In this study, we demonstrate that Nf2/Hippo signalling in the NC and NC‐derived mesenchyme plays an essential role in tongue organogenesis and that continuous expression of *Nf2* in NC‐derived mesenchymal cells is required for the proper tongue formation in shape and size. We provide evidence to suggest that mesenchymal Nf2 regulates Hippo signalling and cell proliferation in a stage‐ and tongue region‐specific manner. Together, the complex and distinct‐from‐other‐organ role of Nf2 in regulating Hippo signalling and cell proliferation during tongue organogenesis bring forward insights into oral/lingual tumourigeneses induced by the alterations of Nf2/Hippo pathway.

## 4 MATERIALS AND METHODS

### 4.1 Animals use and tissue collection

The use of animals was approved by the Institutional Animal Care and Use Committee at the University of Georgia and St Jude Children's Research Hospital. The study was performed in compliance with the National Institutes of Health Guidelines for the care and use of animals in research. The animals were maintained in the animal facilities in the Department of Animal and Dairy Science at the University of Georgia.

C57BL/6 wild type (WT) mice were purchased from The Jackson Laboratory (Stock No: 000664). The *Nf2* floxed allele (fx) was provided by Inserm.^62^ *Wnt1*‐*Cre* (Stock No: 007807, Jackson Laboratory) transgene and *Nf2* fx are both located in Chromosome 11. The recombined allele of *Wnt1*‐*Cre*/ *Nf2^fx^* ^/+^ was generated in Dr. Xinwei Cao's laboratory in St Jude Children's Research Hospital.^35^ The conditional (or tissue‐specific) knockout (cKO) of *Nf2* was generated by breeding *Wnt1*‐*Cre*/*Nf2^fx^* ^/+^ mice with *Nf2^fx^* ^/^ *^fx^*. Cre‐negative littermates served as controls.

Pregnant mice were euthanized with CO_2_ followed by cervical dislocation. Embryos (E10.5–E18.5) were dissected from the uterus under a microscope. E0.5 was designated as noon of the day on which the dam was positive for the vaginal plug. The stages of embryos were confirmed by the development of multiple organs. E10.5 branchial arches (BAs) and various stages of tongues were collected and processed for different analyses. To collect tissues for BrdU immunoreactions, BrdU (B5002, Sigma, St. Louis, MO) was dissolved in Dulbecco's phosphate‐buffered saline (DPBS, Cytiva, Marlborough, MA) at 10 mg/ml and injected intraperitoneally at a single dose of 100 mg/kg 2 hr before embryo collection.

The following primers were used for genotyping: 5'‐CTTCCCAGACAAGCAGGGTTC‐3’ and 5'‐GAAGGCAGCTTCCTTAAGTC‐3’ for *Nf2* fx (~442 bp) and WT (~305 bp) fragments; 5'‐GAAGGCAGCTTCCTTAAGTC‐3’ and 5'‐CTCTATTTGAGTGCGTGCCATG‐3’ for the deleted allele (338 bp) driven by *Wnt1*‐*Cre*; 5′‐TCCAATTTACTGACCGTACACC‐3′ and 5'‐CGTTTTCTTTTCGGATCC‐3’ for the *Cre* gene product (~372 bp).

### 4.2 Immunohistochemistry on sections

Tongue tissues were carefully dissected from the mandible and fixed in 4% paraformaldehyde (PFA) in 0.1 M phosphate‐buffered saline (PBS) at 4°C for 2 hr. PFA‐fixed tissues were cryoprotected in 30% sucrose in 0.1 M PBS at 4°C for at least 24 hr, embedded in O.C.T. compound (#23730571; Fisher Scientific, Waltham, MA), and rapidly frozen for cryostat sectioning at 10 μm in thickness for immunohistochemistry.

Tongue sections were air‐dried at room temperature for 1 hr and rehydrated in 0.1 M PBS. Blocking of non‐specific staining was carried out by incubation with 10% normal donkey serum in 0.1 M PBS containing 0.3% Triton ×‐100 (×100, Sigma Aldrich, St. Louis, MO) at room temperature for 30 min. Then, the sections were incubated with primary antibodies (Table 1) in the carrier solution (1% normal donkey serum, 0.3% Triton ×‐100 in 0.1 M PBS) at 4°C for overnight. Sections without a primary antibody treatment were used as negative control. Following three rinses in 0.1 M PBS, sections were incubated with Alexa Fluor® 488‐ or 647‐conjugated secondary antibody (1:500, Invitrogen, Eugene, OR) in carrier solution at room temperature for 1 hr. Following rinses with 0.1 M PBS, sections were counterstained with DAPI (200 ng/ml in PBS, D1306; Life Technologies, Carlsbad, CA) at room temperature for 10 min. After thorough rinsing in 0.1 M PBS, sections were air‐dried and cover‐slipped with Prolong^®^ Diamond antifade mounting medium (P36970; Fisher Scientific, Waltham, MA). The sections were examined under a fluorescent light microscope (EVOS FL, Life Technologies, Carlsbad, CA) and then photographed using a laser scanning confocal microscope (Zeiss LSM 710, Biomedical Microscopy Core at the University of Georgia).

**TABLE 1:** Primary antibodies used for immunohistochemistry or western blot

Primary antibody	Source (catalogue number, company)	Dilution
Rabbit anti‐p‐Yap	#4911, Cell Signaling Technology, Danvers, MA	1:5000
Rabbit anti‐Yap	#4912S, Cell Signaling Technology, Danvers, MA	1:5000
Rabbit anti‐Nf2	SC−332, Santa Cruz Biotechnology, Inc, Dallas, TX	1:500
Mouse anti‐GAPDH	G8795, Sigma Aldrich, St Louis, MO	1:10,000
Goat anti‐β‐actin	Ab8229, Abcam, Cambridge, United Kingdom	1:10,000
Rat anti‐BrdU	MCA2060, Bio‐Rad, Hercules, CA	1:500
Goat anti‐E‐cadherin	AF748, R&D systems, Minneapolis, MN	1:1000
Rabbit anti‐Krt5	PVB−160P, Covance, Emeryville, CA	1:5000

### 4.3 Scanning electron microscopy

E10.5–E18.5 tongue primordia/tongues from *Wnt1*‐*Cre*/*Nf2^cKO^* mutants and *Cre* ^−^/*Nf2^fx^* ^/^ *^fx^* littermate controls were fixed in 2.5% glutaraldehyde (#75520; Electron Microscopy Science, Hatfield, PA) and 4% PFA in 0.1 M PBS (pH 7.3) at 4°C for 24 hr. After rinsing three times in 0.1 M PBS, tissues were post‐fixed in a sequence of 1% O_S_O_4_ (#19150, Electron Microscopy Science, Hatfield, PA) in 0.1 M PBS, 1% tannic acid (#16201, Sigma Aldrich, St. Louis, MO) in MQ‐H_2_O and 1% O_S_O_4_ in MQ‐H_2_O on ice for 1 hr each. Dehydration was performed in an ascending series of ethanol (35%, 50%, 70%, 90%, 100%) and hexamethyldisilazane (HMDS, #440191; Sigma Aldrich, St. Louis, MO) at room temperature (three changes in each solution, 1 hr each). Tongue tissues were slowly air‐dried in a fume hood and mounted on specimen stubs, sputter coated with gold/palladium (Leica Gold/Carbon coater; Georgia Electron Microscope Core Facility, University of Georgia) and imaged using a scanning electron microscope (FEI Teneo FE‐SEM; Georgia Electron Microscope Core Facility, University of Georgia).

### 4.4 Quantification and measurements

SEM images of E10.5–E18.5 tongue primordia/tongues from *Wnt1*‐*Cre*/*Nf2^cKO^* mutants and *Cre* ^−^/*Nf2^fx^* ^/^ *^fx^* littermate controls (*n* = 3, each group) were used to measure the width of anterior and posterior tongues, and length of the oral tongue using NIH Image‐J software. The width of the anterior oral tongue at 1/3 of length from tip was considered as the anterior tongue width. The width of the widest region where oral tongue connects to the mandible was considered as the width of the posterior oral tongue (posterior width). The distance between the anterior tongue tip and the posterior edge of the circumvallate papilla was considered as the length of the oral tongue (oral tongue length).

Quantitative analyses were made to obtain the number of BrdU^+^ cells per unit area (mm^2^) on E10.5 BAs and E12.5, E15.5, E18.5 tongues of *Wnt1*‐*Cre*/*Nf2^cKO^* and *Cre* ^−^/*Nf2^fx^* ^/^ *^fx^* littermate controls. BrdU immunoreacted serial sections of BAs/tongues from *Wnt1*‐*Cre*/*Nf2^cKO^* mutants and *Cre* ^−^/*Nf2^fx^* ^/^ *^fx^* littermate controls (n=3 each group at each stage) were thoroughly analysed under a fluorescent light microscope (EVOS FL, Life Technologies, Carlsbad, CA). Single‐plane laser scanning confocal photomicrographs were taken from every other section using a scanning confocal microscope (Zeiss LSM 710, Biomedical Microscopy Core at the University of Georgia). BrdU^+^ cells were counted in a unit area (mm^2^) of BAs or the E12.5, E15.5, E18.5 tongues (*n* = 3).

### 4.5 In situ hybridization

Tongues and BAs of *Wnt1*‐*Cre*/*Nf2^cKO^* mutants and *Cre* ^−^/*Nf2^fx^* ^/^ *^fx^* littermate controls were dissected in 0.1 M PBS, fixed with 4% PFA at 4°C for 24 hr. PFA‐fixed tissues were washed three times in 0.1 M PBS and dehydrated through ascending series of methanol (35%, 50%, 70%, 90%, 100%) and stored in 100% methanol at −20°C. Tissues were rehydrated using 0.1 M PBS and in situ hybridization was performed as described previously.^63^ Riboprobes for Nf2 was generated by in vitro transcription using primers 5’‐GAGGCAATTAACCCTCACTAAAGGTTGGCTGAAAAGGCTCAGAT‐3’, 5’‐GAGTAATACGACTCACTATAGGGCCCGCTCTTTGAGTTTCAAG‐3’ and a dig‐UTP labelling mix (Roche, Basel, Switzerland) following the manufacturer's specifications.

### 4.6 RNA extraction and quantitative reverse transcriptase PCR

Epithelium and mesenchyme were separated from the E12.5 *Cre* ^−^/*Nf2^fx^* ^/^ *^fx^* littermate control and *Wnt1*‐*Cre*/*Nf2^cKO^* mutant tongues as described previously.^64^ Briefly, dissected E12.5 *Cre* ^−^/*Nf2^fx^* ^/^ *^fx^* littermate control and *Wnt1*‐*Cre*/*Nf2^cKO^* tongues were incubated in a mixture of 1 mg/ml Collagenase A (#10103578001; Roche Diagnostics, Basal, Switzerland) and 2.5 mg/ml Dispase II (#4942078001; Roche Diagnostics, Basal, Switzerland) enzymes at 37°C for 30 min. After a thorough rinse in 0.1 M PBS, epithelial sheets were removed from the mesenchyme. Separated mesenchyme and epithelia were then transferred to Trizol (#15596018; Life Technologies, Carlsbad, CA) solution for RNA extraction using an RNA extraction kit (#74136; Qiagen, Hilden, Germany). A total of nine epithelial and mesenchymal tissues were used for RNA extraction (three replicates each with three tissues pooled together). RNA concentrations were measured using Nanodrop 8000 spectrophotometer (Nanodrop, Thermo Scientific, Waltham,). Complementary DNA (cDNA) was converted from the extracted RNA using SuperScript™ First‐Strand Synthesis System (#11902018; Fisher Scientific, Waltham, MA).

Quantitative RT PCR was conducted using the cDNA to analyse the expression of Yap target genes using the primers in Table 2. Changes of gene expression levels in tongue epithelium and mesenchyme of *Wnt1*‐*Cre*/*Nf2^cKO^* mutant and *Cre* ^−^/*Nf2^fx^* ^/^ *^fx^* littermate control groups were presented as mean ± standard deviation (X ± SD; *n* = 3) of 2^−ΔCT^ values.

**TABLE 2:** Primer sequences used for the quantitative reverse transcriptase PCR

Gene	Primer sequence
*Amotl1*	Forward 5′ CAGAGGAGAACCGTGTGCTTCA 3′ Reverse 5′ TTGCCTTGTCCAGAGACTCACG 3′
*Ankrd1*	Forward 5′ GCTTAGAAGGACACTTGGCGATC 3′ Reverse 5′ GACATCTGCGTTTCCTCCACGA 3′
*Areg*	Forward 5′ GCAGATACATCGAGAACCTGGAG 3′ Reverse 5′ CCTTGTCATCCTCGCTGTGAGT 3′
*Axl*	Forward 5′ GGTGTTTGAGCCAACCGTGGAA 3′ Reverse 5′ GCCACCTTATGCCGATCTACCA 3′
*Birc5*	Forward 5′ CCTACCGAGAACGAGCCTGATT 3′ Reverse 5′ CCATCTGCTTCTTGACAGTGAGG 3′
*Ctgf*	Forward 5′ CCACCCGAGTTACCAATGAC 3′ Reverse 5′ GTGCAGCCAGAAAGCTCA 3′
*Cyclin D1*	Forward 5′ GAC GGC GTC AAA TAT GTC CT 3′ Reverse 5′ CTG GAG AGT GAC AGC ATG GA 3′
*Cyr61*	Forward 5′ GTGAAGTGCGTCCTTGTGGACA 3′ Reverse 5′ CTTGACACTGGAGCATCCTGCA 3′
*Nf2*	Forward 5′ GGGGAAGGACCTGTTTGATT 3′ Reverse 5′ GACAGCATATGACGCCAAGA 3’

### 4.7 RNA sequencing

Extracted and qualified RNA from E14.5 *Wnt1*‐*Cre*/*Nf2^cKO^* and Cre^−^/Nf2^fx/fx^ tongues were processed for the generation of cDNA libraries for sequencing on a NextSeq 500 system (Illumina) (Georgia Genomics Facility, University of Georgia). Sequenced reads were used for mapping to the UCSC (http://genome.ucsc.edu/) *Mus musculus* reference genome using Tophat package with default parameters, and the aligned reads were used by Cufflinks to measure the relative abundances of transcripts (fragments per kilobase million, FPKM). The differentially expressed genes were displayed only for comparisons with a ‘q value’ <0.01 and status marked as ‘OK’ in the Cuffdiff output, which will be further used for GO Terms and KEGG pathway analysis based on our previous publication.^65^

### 4.8 Western blot

Proteins in BAs and tongues of *Wnt1*‐*Cre*/*Nf2^cKO^* mutants and *Cre* ^−^/*Nf2^fx^* ^/^ *^fx^* littermate controls were extracted using radioimmunoprecipitation assay (RIPA) buffer (1% NP‐40, 150 mmol/L NaCl, 50 mmol/L Tris‐HCI, 0.5% sodium deoxycholate, 0.1% SDS, 1 mmol/L EDTA, pH 7.4). The concentration of extracted proteins was determined using Pierce™ BCA protein assay kit (#23225; Thermo Fisher Scientific) and Synergy™ 4 microplate reader (#7161000; BioTek Instruments, Winooski, VT). An amount of total protein from posterior and anterior oral tongue mesenchyme (50 µg for E12.5, E15.5; 20 µg for E18.5) was loaded into each lane. Sodium dodecyl sulphate–polyacrylamide gel electrophoresis (SDS‐PAGE) was used to resolve the protein bands and transferred to the nitrocellulose membrane. Non‐specific binding was blocked using blocking buffer containing 3% bovine serum albumin (A‐420–100; Gold Biotechnology, St Louis, MO) in Tris‐buffered saline and Tween‐20 buffer (TBST) buffer (20 mM Tris pH 7.5, 150 mM NaCl, 0.1% Tween 20) at room temperature for 1 hr. The membranes were incubated with primary antibodies (Yap, p‐Yap, Gapdh and β‐actin; Table 1) in blocking buffer at 4°C for 24 hr. Following three rinses in TBST (10 min each), membranes were incubated with Alexa Fluor 647‐conjugated secondary antibodies in blocking buffer at room temperature for 2 h. ChemiDocMP Imaging System (Bio‐Rad ChemiDoc, Hercules, CA) was used to image the Western blot bands.

### 4.9 Statistical analysis

Student's *t*‐test was used to analyse the statistical significance of differences between *Wnt1*‐*Cre*/*Nf2^cKO^* mutants and *Cre* ^−^/*Nf2^fx^* ^/^ *^fx^* littermate controls for the indices below: the oral tongue length, anterior and posterior tongue widths, Western blot band intensities of Yap, p‐Yap, and Gapdh. Two‐way analyses of variance (ANOVA) followed by Fisher's LSD analyses were used to compare BrdU^+^ cells per unit area (mm^2^) in individual BAs and tongues between *Wnt1*‐*Cre*/*Nf2^cKO^* mutants and *Cre* ^−^/*Nf2^fx^* ^/^ *^fx^* littermate controls. A *p*‐value <0.05 was taken as statistically significant.

## 5 CONCLUSION

Our data indicate a region‐ and stage‐specific role of Nf2 in NC‐derived tongue mesenchyme in regulating Hippo signalling and cell proliferation, which is in distinction from many other organs.

### 5.1 NF2/Hippo signalling shapes tongue organ

Schematic diagram to represent the stage‐ and region‐specific roles of Nf2‐mediated Hippo signalling in the mesenchyme during tongue organogenesis. Nf2 deletion in neural crest‐derived tongue mesenchyme resulted in distinct alterations of Yap level and cell proliferation causing non‐proportionately larger tongue (macroglossia) during the early stages of tongue development (i.e. E12.5–E13.5) and microglossia during the later stages of tongue development (i.e. E15.5–E18.5).

## CONFLICT OF INTEREST

All authors have no conflicts of interest to declare.

## AUTHOR CONTRIBUTIONS

MI, GC and H‐XL contributed to the experimental design; MI, GC, WY, ZW and H‐XL conducted the experiment; MI, GC and H‐XL did the data collection and analysis; MG, XC and H‐XL provided the material; MI and H‐XL drafted and edited the manuscript.

## Data Availability

The data that support the findings of this study are available from the corresponding author upon reasonable request.
